# Weather-Related Flood and Landslide Damage: A Risk Index for Italian Regions

**DOI:** 10.1371/journal.pone.0144468

**Published:** 2015-12-29

**Authors:** Alessandro Messeri, Marco Morabito, Gianni Messeri, Giada Brandani, Martina Petralli, Francesca Natali, Daniele Grifoni, Alfonso Crisci, Gianfranco Gensini, Simone Orlandini

**Affiliations:** 1 Interdepartmental Centre of Bioclimatology, University of Florence, Piazzale delle Cascine 18, 50144 Florence, Italy; 2 Department of Agrifood Production and Environmental Sciences, DISPAA, University of Florence, Piazzale delle Cascine 18–50144 Florence, Italy; 3 Institute of Biometeorology, National Research Council—Via Giovanni Caproni 8, 50145 Florence, Italy; 4 Consortium LaMMa (Laboratory of Monitoring and Environmental Modelling for the Sustainable Development) Via Madonna del Piano 10, 50019 Sesto Fiorentino (Florence), Italy; 5 Clinica Medica e Cardiologia, University of Florence-Viale Morgagni 85, 50134 Florence, Italy; University of Vigo, SPAIN

## Abstract

The frequency of natural hazards has been increasing in the last decades in Europe and specifically in Mediterranean regions due to climate change. For example heavy precipitation events can lead to disasters through the interaction with exposed and vulnerable people and natural systems. It is therefore necessary a prevention planning to preserve human health and to reduce economic losses. Prevention should mainly be carried out with more adequate land management, also supported by the development of an appropriate risk prediction tool based on weather forecasts. The main aim of this study is to investigate the relationship between weather types (WTs) and the frequency of floods and landslides that have caused damage to properties, personal injuries, or deaths in the Italian regions over recent decades. In particular, a specific risk index (WT-FLARI) for each WT was developed at national and regional scale. This study has identified a specific risk index associated with each weather type, calibrated for each Italian region and applicable to both annual and seasonal levels. The risk index represents the seasonal and annual vulnerability of each Italian region and indicates that additional preventive actions are necessary for some regions. The results of this study represent a good starting point towards the development of a tool to support policy-makers, local authorities and health agencies in planning actions, mainly in the medium to long term, aimed at the weather damage reduction that represents an important issue of the World Meteorological Organization mission.

## Introduction

The frequency of natural hazards has been increasing in the last decades in Europe, and more specifically in the Mediterranean regions, due to climate change [1, 2, 3, 4, 5, 6, 7]. Heavy precipitation events can lead to disasters through interaction between exposed and vulnerable people and the natural systems [8, 9, 10]. In particular, floods and landslides are considered important natural disasters with significant effects in terms of the number of people affected and the economic losses [11]. The impacts of floods and landslides are determined not just by their magnitude, but also by human and societal choices related to infrastructures, behaviors and other factors [12, 13, 14, 15, 16]. The immediate and direct impacts of these events on human health include drowning, heart attacks, various injuries, and hypothermia. Furthermore, indirect impacts, such as infections, water-borne infectious diseases, mental health disorders, respiratory diseases and allergies in both the medium and long term, should also be considered as significant effects [17, 18, 19, 20, 21]. In Italy, it has been estimated that over 68% of the municipalities are at high hydrogeological risk [22] and in recent decades, intense rainfall events have caused severe disruptions [23, 24].

In addition, the risk of natural disasters in Italy is still rising due to the increased population density, progressive urbanization, abandonment of mountainous areas, unauthorized buildings, ongoing deforestation, and lack of maintenance of the slopes and waterways [25]. The effects of floods and landslides are often underestimated because of the lack of an inventory system and damage cataloging [26, 27]. Consequently, investigation of the historical effects of these natural hazards is fundamental for the temporal reconstruction of the events and for assessing their potential frequency and consequent effects [28, 29]. For this purpose, the “Inventory of areas affected by landslides and floods in Italy Project” (AVI), commissioned by the Department of Civil Protection and created by the National Research Council, has enabled the development of a detailed Italian database of surveys and damage caused by landslides and floods, from the early 1900s until today.

The increase in the frequency of these events calls for prevention planning to preserve human health and reduce economic losses. Prevention should mainly be carried out with more adequate land management, also supported by the development of an appropriate risk prediction tool based on weather forecasts[30].

However, warnings for severe meteorological events potentially able to cause landslides and floods are only issued a few hours before the event (now casting) or with 2–3 days' notice. This is because weather forecast services only have high reliability in the short-medium term. Nonetheless, the safety operation of major risk situations often requires several days or even weeks' preparedness and a few days warning might not be sufficient.

Several innovative warning tools would therefore be very useful for this purpose. Seasonal climate forecast models are used increasingly across a range of application sectors and could be implemented in new procedures to support health prevention. These models have been developed from ensembles of integrations of numerical climate models [31] and offer good probabilistic reliability. Weather-circulation type (WT) classification could represent a useful tool for improving the reliability of seasonal weather forecasts. The application of WT is a well-established approach in synoptic and applied climatology, ranging from support for weather forecasting to climate model validations or downscaling [32, 33, 34, 35].

The main aim of this study is to investigate the relationship between WT and the frequency of floods and landslides that have caused damage to properties, personal injuries, or deaths in the Italian regions over recent decades. In addition, specific risk indexes (WT-FLARI) have been developed for each WT at a national and regional scale.

The result of this work represent a good starting point towards the development of a tool to support policy-makers, local authorities and health agencies in planning actions, mainly in the medium to long term, aimed at the reduction of disasters. Disasters reduction represents an important issue of the World Meteorological Organization mission [36]. Actions to be taken on the eve on an emergency will nevertheless still predominantly managed through the use of deterministic models able to better locate the phenomena, but in the medium to long term (months), the WT approach could instead make possible a better use of seasonal forecasts, which, although in gradual improving, will unlikely be able to provide deterministic forecasts, on the contrary could provide useful information on the prevailing WT.

## Materials and Methods

**Fig 1:**
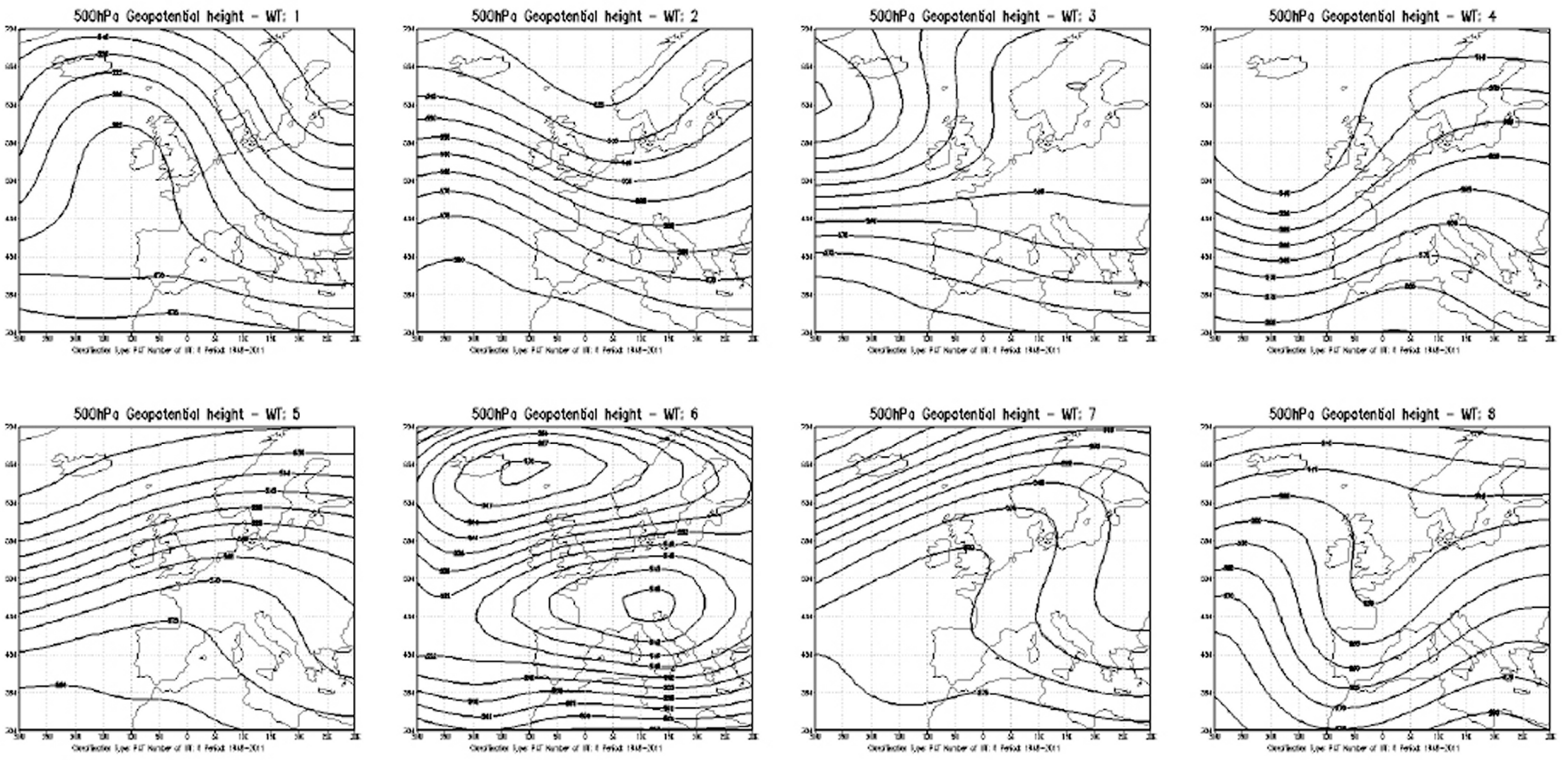
500hPa geopotential height for each weather types (WT) classified by LAMMA-IBIMET for the period 1948–2011.

The study was carried out with an analysis of the connection between atmospheric circulation types and the damage caused by landslides and floods in Italy during the 1948–2003 period. The analysis was based on the following datasets:

1. The NCEP/NCAR Reanalysis 1 (NCEP1) global grilled dataset [37, 38] was used to create a national weather type classification for the investigated period, 1948–2003. The NCEP1 data, on 2.5°x2.5°, are available at http://www.cdc.noaa.gov/cdc/reanalysis.
2. A database of the most common weather and circulation types (WTs) in Europe by using the cost733class software package to create, compare, visualize and evaluate weather and circulation-type classifications [34,39]. Data sources are freely available at http://cost733.met.no/. The NCEP reanalysis data were utilized on a latitude-longitude grid (30N-70N, 30W-30E) and then the Principal Component in T mode (PCT) was implemented, using a geopotential height at 500htp [40, 41, 42, 43]. According to Philipp et al [34], there is no clear statistical reason to prefer any of the classified methods of COST733 software package. Consequently no new classification was created and implemented specifically for this study.Therefore, we used an existing classification already applied both for operative-forecast purpose (LAMMA-IBIMET) that for scientific study [34, 35]. The weather-type classification was set to eight classes as representative of the main circulation types prevailing over the Italian Peninsula (Fig 1, Table 1).
3. A database of landslide and flood events that caused damage created by the National Group for Prevention of Hydrological Hazards (GNDCI) of the National Research Council (CNR) with regard to the AVI Project commissioned by the Department of Civil Protection. The AVI Inventory is a homogeneous and updated archive with a detailed spatial representation of landslides and floods. This is an important tool for hazard-risk analysis and land-use planning. In particular, landslides and floods that caused damage from the early 1900s until today were collected and organized. Furthermore, within this study, the “Inventory of Landslide Phenomena in Italy” [44] was taken into account. However, in this study only years with more reliable data starting from late forties were considered (http://avi.gndci.cnr.it/welcome_en.htm). All data used in this work are available on https://github.com/meteosalute/weather_landslide.

**Table 1:** The most common Weather Types (WT) in Europe and prevailing circulation in Italy.

WT number	Characteristics of circulation
1	Marked northward expansion of the Azores anticyclone with blocked anticyclonic circulation over the North Atlantic and northerly winds over Italy
2	Moderate northward expansion of the Azores anticyclone with cyclonic circulation over south Scandinavia and north-westerly winds over Italy
3	Marked cyclonic circulation over Iceland with anticyclonic circulation over northern central Europe accompanied with increased precipitation over Italy, generated by intermittent Atlantic perturbations
4	Cyclonic circulation over the North Atlantic and cyclonic circulation over west Mediterranean Europe and central Mediterranean Europe with decreased precipitations over central Mediterranean Europe
5	Cyclonic circulation over the north-west Atlantic with marked anticyclonic circulation over west Mediterranean Europe and central Mediterranean Europe, inducing warm and dry conditions over Italy
6	Anticyclonic circulation over Iceland and cyclonic circulation over central Europe, with higher precipitation over Tuscany by intrusions of artic and polar continental air
7	South westerly flow over the North Atlantic with ridging over the British Isles towards Scandinavia, with easterly wind over central Mediterranean Europe resulting in very cold dry conditions
8	Cyclonic circulation over west Europe with a ridge over the eastern Mediterranean

Flood and landslide events were organized and processed separately for each region, in order to obtain a detailed overview of the number of events per day from 1948 to 2003. Therefore, a comprehensive database for each Italian region was created on a daily basis including the occurrence of WTs and of landslide/flood events which then were aggregated on a seasonal and annual basis and expressed as relative frequency for each WT.

A non-parametric test [45] was used to compare the number of events for each WT between seasons in the period 1948–2003.

These events together with exposure and vulnerability layers, represented by the population density (inhabitants per km^2^), river surface (km) and non-plain surface (km^2^) were used as input data to create a specific WT-related Flood and LAndslide Risk Index (WT-FLARI) calibrated at both annual and seasonal level.

**Fig 2:**
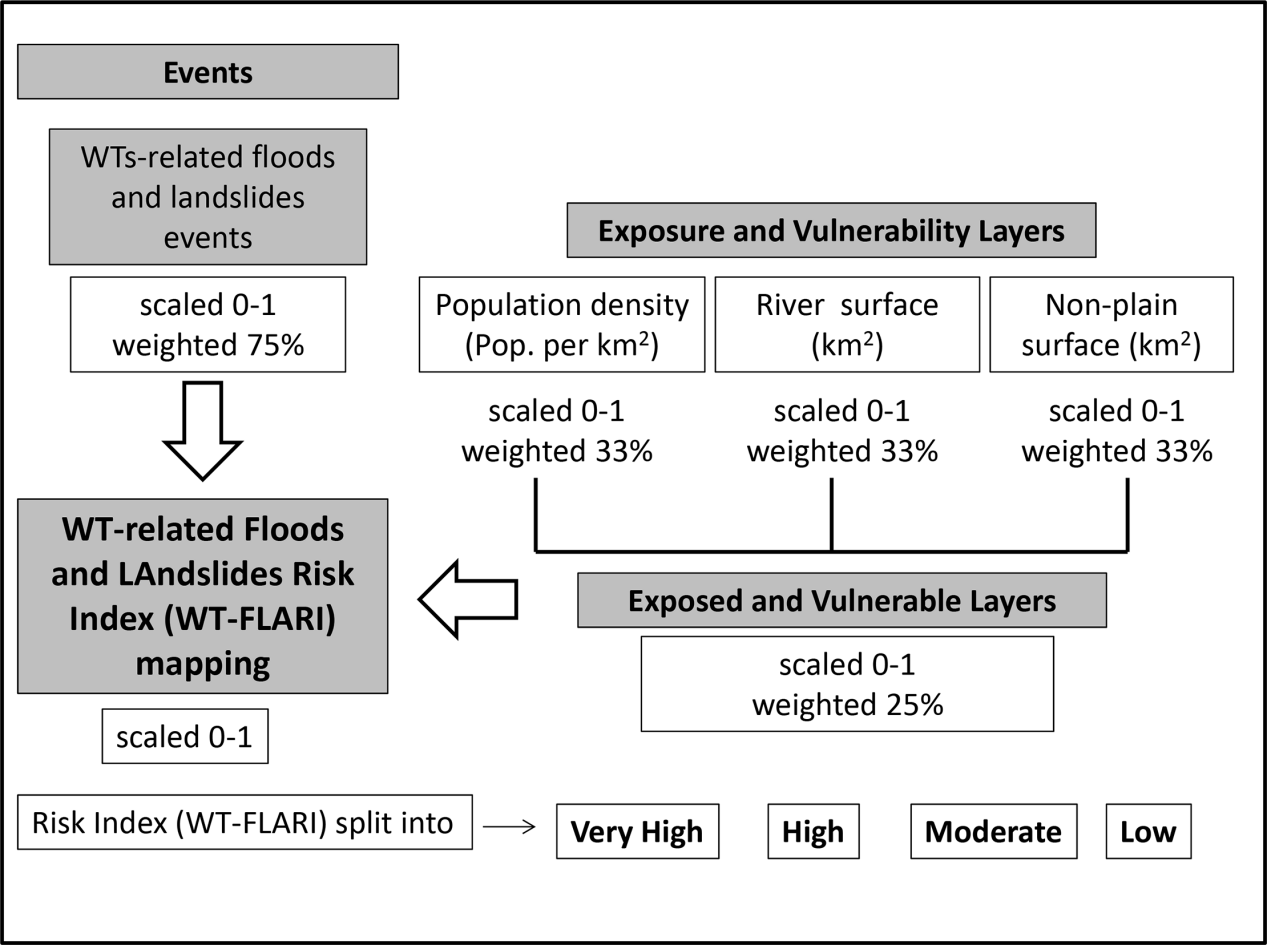
Work-flow of the Weather Type-related Floods and Landslides Risk Index (WT-FLARI) assessment.

The study design focused on “Crichton’s Risk Triangle” hazard-risk assessment methodology [46] developed in the field of the ASCCUE (Adaptation Strategies for Climate Change in the Urban Environment) project. The workflow of the hazard risk analysis employed to develop the final mapping of the WT-FLARI is shown (Fig 2).

The risk concept is represented by harmful consequences on human health and the territory resulting from the interaction between three components that form a triangle: hazard, exposure, and vulnerability. The risk is defined as a function of these three components.

More specifically, a normalization procedure, followed by a weighted-layer combining procedure was applied for each Italian region. By means of the normalization procedure events occurrence, exposure and vulnerability layers were rescaled from 0 to 1. The following step was the combination of the normalized layers through a weighting procedure. The exposure and vulnerability layers, (Table 2), were renormalized in a single “exposed and vulnerable layer” where each variable was weighted at 33.3%.

**Table 2:** Population density, river surface and non-plain surface for each Italian Region.

**Country**	**Population density (Pop. per km** ^**2**^ **)**	**River surface(km)**	**Non-plain surface (km** ^**2**^ **)**
Abruzzo	121	4671	10831
Basilicata	57	5550	9267
Calabria	129	9504	15221
Campania	422	6147	11675
Emilia Romagna	195	10587	11720
Friuli Venezia Giulia	155	4005	4866
Lazio	322	9464	13803
Liguria	289	3943	5416
Lombardia	410	10289	12623
Marche	164	5594	9401
Molise	70	2414	4460
Piemonte	172	15077	18684
Puglia	207	4395	9145
Sardegna	68	15104	19641
Sicilia	194	11122	22164
Toscana	161	15098	21056
Trentino Alto Adige	76	7355	13605
Umbria	105	5214	8464
Veneto	265	7553	8025

Following, the “exposed and vulnerable” layer (weighted at 25%) was combined with the event layer (weighted at 75%) to obtain the final WT-FLARI varying between 0 and 1 (Fig 2). The final annual and seasonal mapping visualization of RI was carried out via identification of specific thresholds indicating the actual ongoing risk. The threshold identification was performed using quantiles, with particular reference to the values over 90% [47]:

- very high risk (RI> 99%)
- high risk (95%<RI< 99%)
- medium risk (90%<RI< 95%)
- low risk (RI< 90%).

Extreme-value statistics describe the probabilities associated with a quantity exceeding a given threshold value according to a Poisson distribution [48, 49, 50, 51].

The thresholds were only identified for the annual risk index and then applied to a seasonal level instead, in order to obtain a risk assessment that also considered the weight given to each WT in each season.

## Results

### Weather type distributions

The weather configuration characterized by the presence of the Azores High Pressure over the Mediterranean Basin (WT5) showed the highest annual WT frequency (25%, **Table 3**), followed by WT2 (22%) characterized by partial displacement of the Azores High Pressure to Northern Atlantic Ocean resulting in the flow of maritime polar air masses towards Central Europe and partly in the Mediterranean basin.

**Table 3:** Seasonal distribution of weather types and flood and landslide events from 1948 to 2003.

	WT1		WT2		WT3		WT4		WT5		WT6		WT7		WT8	
WT Frequency	10%		22%		11%		12%		25%		0%		11%		9%	
	N.	E	N.	E	N.	E	N.	E	N.	E	N.	E	N.	E	N.	E
*Season*																
A	506	27	960	158	574	150	511	219	1601	285	1	0	458	123	480	504
Sp	613	27	989	76	584	37	782	45	958	71	2	0	565	60	659	90
Su	272	25	1381	97	626	29	730	29	1199	100	0	0	601	50	343	136
W	675	70	1073	156	538	83	489	72	1309	99	15	2	550	63	383	119
*ANOVASig*. *E*	p<0.01		p<0.001		p<0.001		p<0.001		p<0.001		p = 0.905		p<0.01		p<0.001	

WT = Weather Type; N = the specific WT frequency; E = number of events related to a specific WT; Su = summer; A = autumn; W = winter; Sp = spring; p = a non parametric method of the analysis of variances [45] was used to compare the number of events for each WT between season.

Annual frequencies ranging from 9% to 12% were observed when WT1, WT3, WT4, WT7 and WT8 were considered. The weather configuration characterized by a large high pressure block in Northern Europe and the Atlantic Ocean with cold air masses in Mediterranean Basin and Central Europe (WT6) showed the lowest frequency with very few cases (18) during the period studied. The predominance of WT2 and WT5 was also confirmed in all seasons (**Table 3**), with frequencies often greater than 20%. In addition, other high frequencies were also observed for the subtropical high pressure (WT4) in summer (14%) and spring (15%), and for WT1 and WT3 in winter (13%) and autumn (11%) respectively.

### WT- related flood and landslide events

Almost half the flood and landslide events occurred in autumn (49%) followed by winter (22%), summer (15%) and springer (14).

A statistical analysis (kruskal wallis test) showed that the weather type significantly discriminate the frequency of events in each season (Autumn P<0.001, Summer P<0.001, Winter P<0.001, Spring P<0.01). In particular WT8 is the weather type that has the highest frequencies in each season (data not shown).

No significant seasonal events variations were observed when WT1 and the rare WT6 were considered (Table 3). However, in these weather conditions the highest frequency of events was observed in winter.

The other WTs always showed significantly higher values in autumn than the other seasons (Table 3). Other significant variations were also observed in winter with significantly higher values than spring and summer when WT2 and WT3 occurred.

In particular, WT8 showed the highest frequency during autumn (34% of autumnal frequencies of events), summer (29%) and spring (22%). During winter the highest frequency was observed when WT2 (23%) occurred, followed by WT8 (18%). Other high frequencies (>15% of seasonal frequencies) were observed in summer for WT5 (22%) and WT2 (21%); in autumn for WT5 (19%) and WT4 (15%); in spring for WT2 (19%) and WT5 (18%).

Several WTs caused damage, especially in northern Italy, while others occurred in central and southern regions (Table 4).

**Table 4:** Percentage of events for each region and for each WTs.

**Region**	**WT1**	**WT2**	**WT3**	**WT4**	**WT5**	**WT6**	**WT7**	**WT8**
Abruzzo	0.24	0.16	0.17	0.08	0.14	5.56	0.46	0.32
Basilicata	0.29	0.48	0.56	0.36	0.39	0	0.60	0.21
Calabria	0.39	0.34	0.65	0.24	0.45	5.56	0.64	0.16
Campania	0.92	1.79	1.68	1.15	1.44	0	1.20	2.31
Emilia Romagna	0.15	0.27	0.30	0.28	0.18	0	0.32	0.86
Friuli Venezia Giulia	0.15	0.32	0.39	0.12	0.10	0	0.18	0.97
Lazio	0.44	0.77	0.43	0.84	0.45	0	0.60	1.77
Liguria	0.29	0.23	0.60	0.84	0.26	0	0.09	1.23
Lombardy	0.05	0.48	0.65	1.07	0.49	0	0.23	2.68
Marche	0.24	0.20	0.17	0.08	0.20	0	0.51	0.38
Molise	0.05	0.07	0.04	0.04	0.06	0	0.05	0.11
Piedmont	0.10	0.27	0.60	0.64	0.47	0	0.37	2.47
Puglia	0.63	0.36	0.43	0.24	0.53	0	0.78	0.59
Sardinia	0.44	0.64	1.12	0.56	0.79	0	0.78	1.39
Sicily	0.73	0.36	0.39	0.44	0.49	0	1.01	0.64
Toscany	0.34	0.61	0.69	0.52	0.28	0	0.37	1.34
Trentino Alto Adige	0.24	0.55	0.47	0.32	0.28	0	0.32	1.45
Umbria	0.44	0.45	0.13	0.12	0.12	0	0.18	0.54
Veneto	0.34	0.59	0.43	0.28	0.28	0	041	1.45

For example, WT8 caused a greater number of events in central and northern Italian regions, particularly Lombardy, Piedmont and Lazio, as well as Campania. Conversely, WT6 and WT7 determined effects, especially in Abruzzo, Calabria, Campania, Apulia, Sardinia and Sicily. Campania showed the highest frequency of flood and landslide events when almost all WTs occurred, with the exception of WT6 and WT8, where the records were held by Calabria and Lombardy respectively. The region which showed the lowest frequency of events for each WTs was Molise.

### Exposure and vulnerability layer distributions

**Fig 3:**
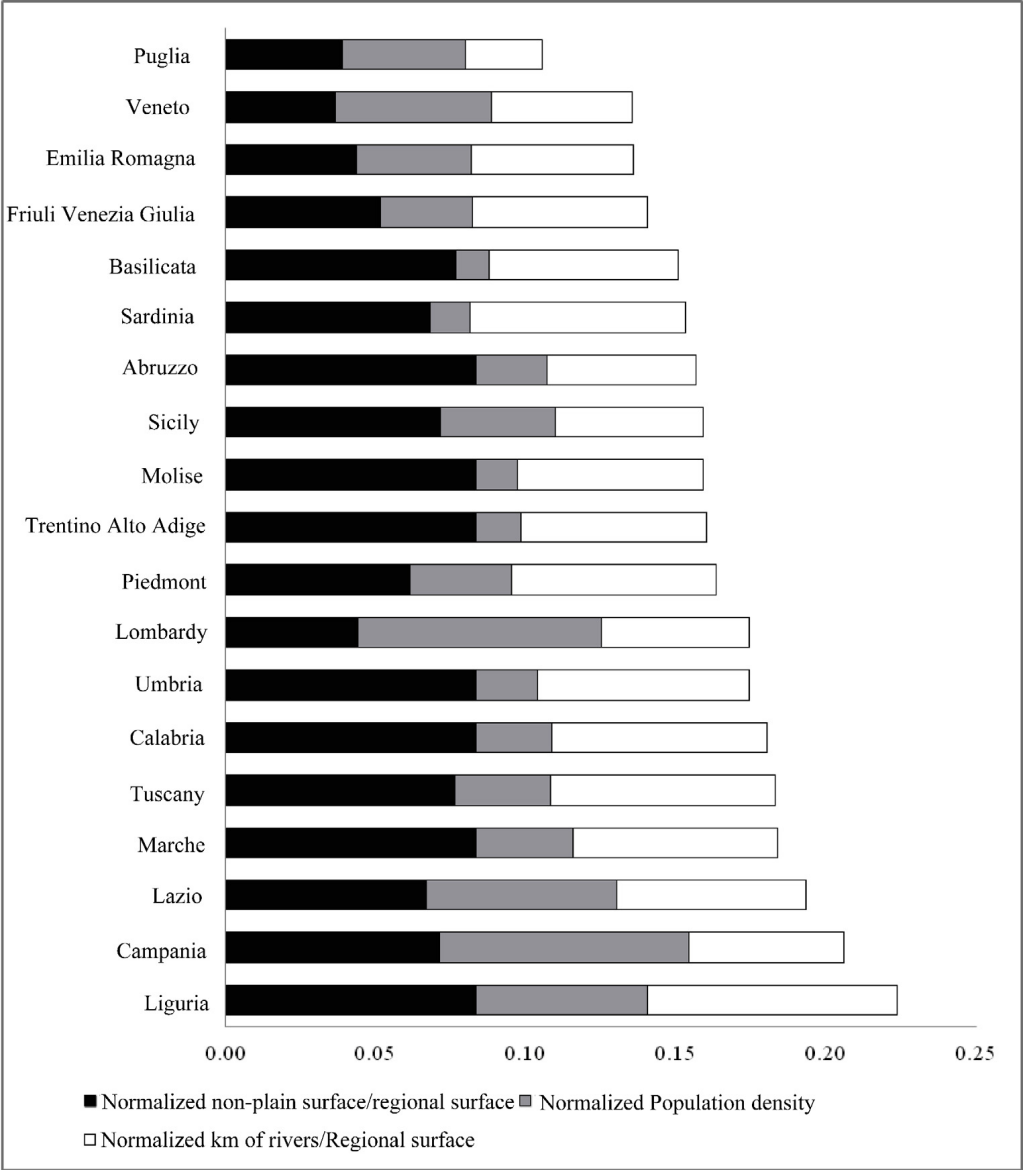
Incidence of normalized features for each Italian region.

Among all regions, Liguria had the highest values of Exposed and Vulnerable Layers (Fig 3), especially due to non-plain surface and the high number of rivers in relation to regional surface. Even Campania showed very high values determined primarily by the high population density. The lowest values were in Apulia, Veneto, Emilia Romagna and Friuli Venezia Giulia where the contribution of the non-plain surface is very low.

### The annual WT-related flood and landslide Risk Index

**Fig 4:**
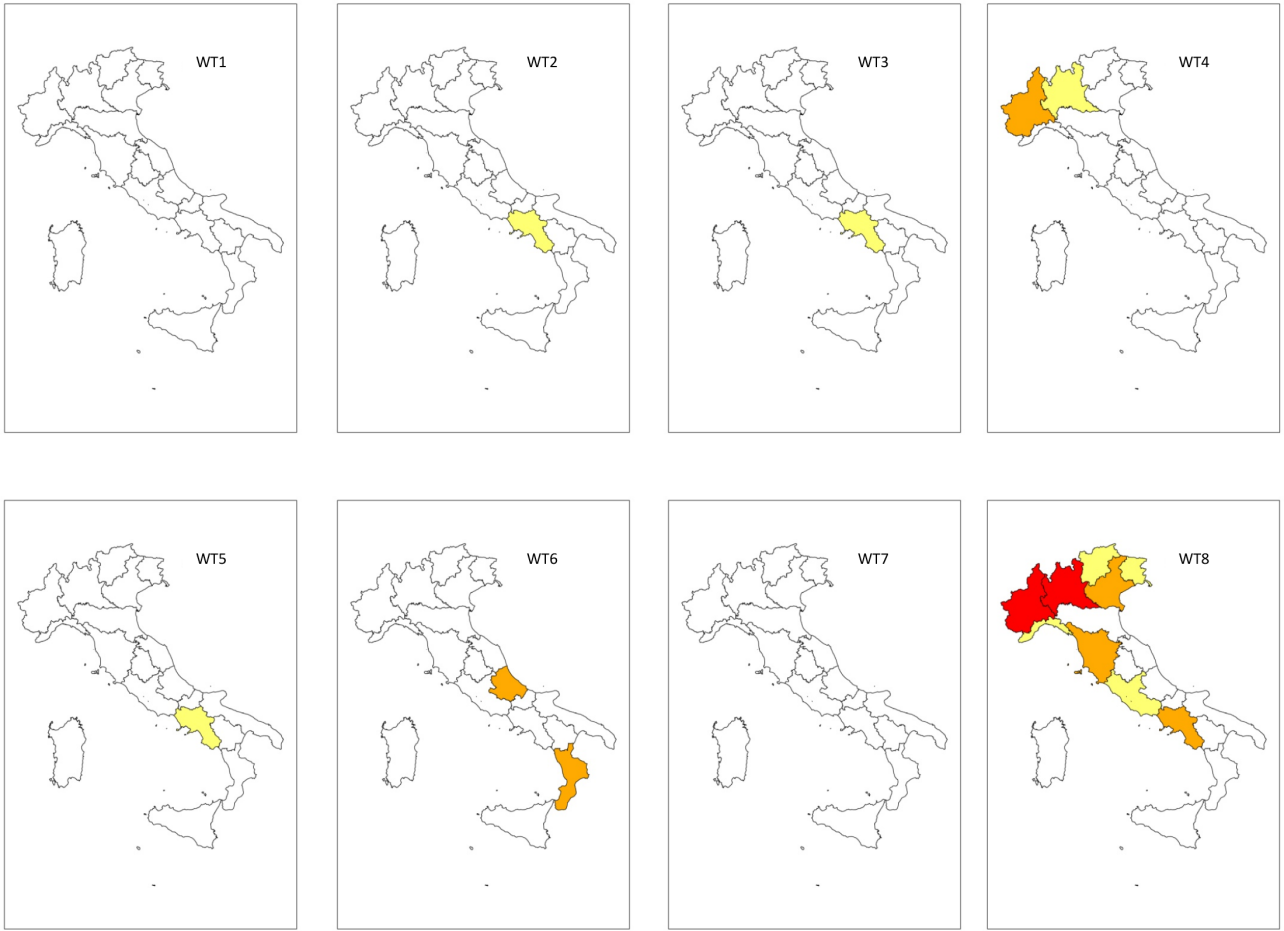
Mapping of the annual WT-related Floods and LAndslides Risk Index (WT-FLARI). WT (Weather Type). WT-FLARI levels: red = Very High (WT-FLARI > 99^th^ perc.); orange = High (95^th^ perc. > WT-FLARI > 99^th^ perc.); yellow = Moderate (95^th^ perc. > WT-FLARI > 90^th^ perc.); white = Low (WT-FLARI < 90^th^ perc.)

The WT8 is the most dangerous meteorological configuration at an annual Italian level (Fig 4).

Piedmont and Lombardy are the regions that reached the “very high” WT-FLARI level (red in Fig 4), followed by Tuscany, Campania and Veneto, which showed the “high” risk level (orange in Fig 4). A “moderate” risk level was observed in Lazio, Trentino Alto Adige and Friuli Venezia Giulia (yellow in Fig 4).

WT2, WT3 and WT5 only showed a “moderate” risk level in Campania.

WT4 determined a “high” risk level in Piedmont, while the risk level was “moderate” in Lombardy. Conversely, WT4 showed a “low” risk level for all central and southern Italian regions which were generally protected by high pressure whenever WT4 occurred.

The very rare WT6 had the greatest effects on southern Italian regions. A “high” risk level (Fig 4) was observed in the Marche and Calabria, which are more exposed to the eastern airflow that often determines a low depression between the Adriatic Sea and the Ionian Sea. On the other hand, there were no risk conditions for WT1 and WT7, which were also characterized by a predominant Eastern circulation.

### Seasonal WT-related flood and landslide Risk Index

**Fig 5:**
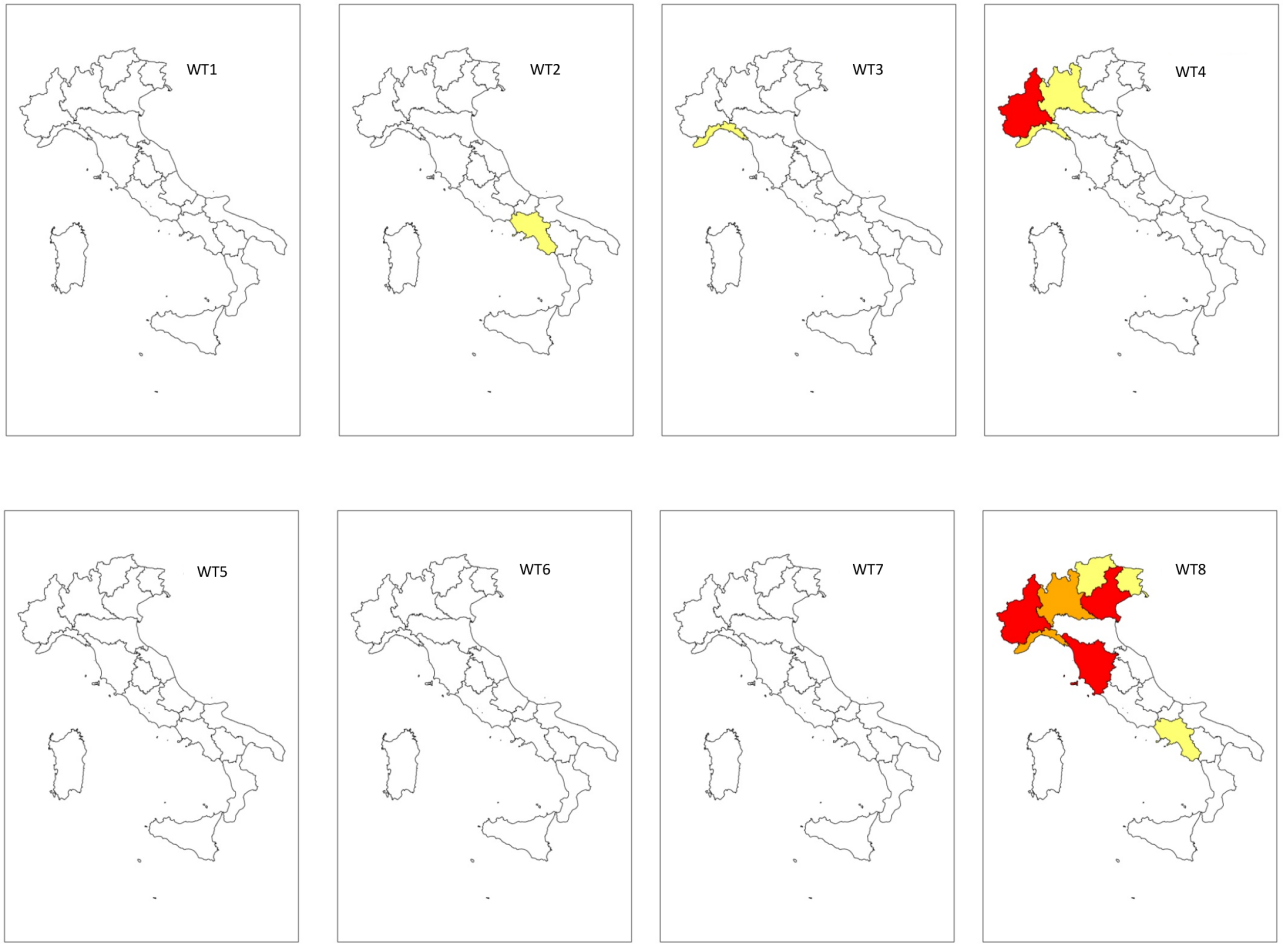
Mapping of the autumn WT-related Floods and LAndslides Risk Index (WT-FLARI). WT (Weather Type). WT-FLARI levels: red = Very High (WT-FLARI > 99^th^ perc.); orange = High (95^th^ perc. > WT-FLARI > 99^th^ perc.); yellow = Moderate (95^th^ perc. > WT-FLARI > 90^th^ perc.); white = Low (WT-FLARI < 90^th^ perc.)

Varying seasonal WT-FLARI patterns were recorded. In autumn (Fig 5), the hazardous risk levels (“Very High” and “High”), which were always associated with WT8 and WT4, prevailed in the northern regions.

In particular, the most dangerous risk level was concentrated in the north (Lombardy and Veneto) and one central region (Tuscany) for WT8, and in the northwest (Lombardy) for WT4.

The “High” risk was only observed in northern regions when WT8 occurred. The “Moderate” risk was prevalently observed in the northeast and associated with WT8, and in one southern region (Campania) for WT8 and WT2. “Moderate” risk was also observed in several northern regions (Liguria and Lombardy) when WT4 and WT3 occurred.

**Fig 6:**
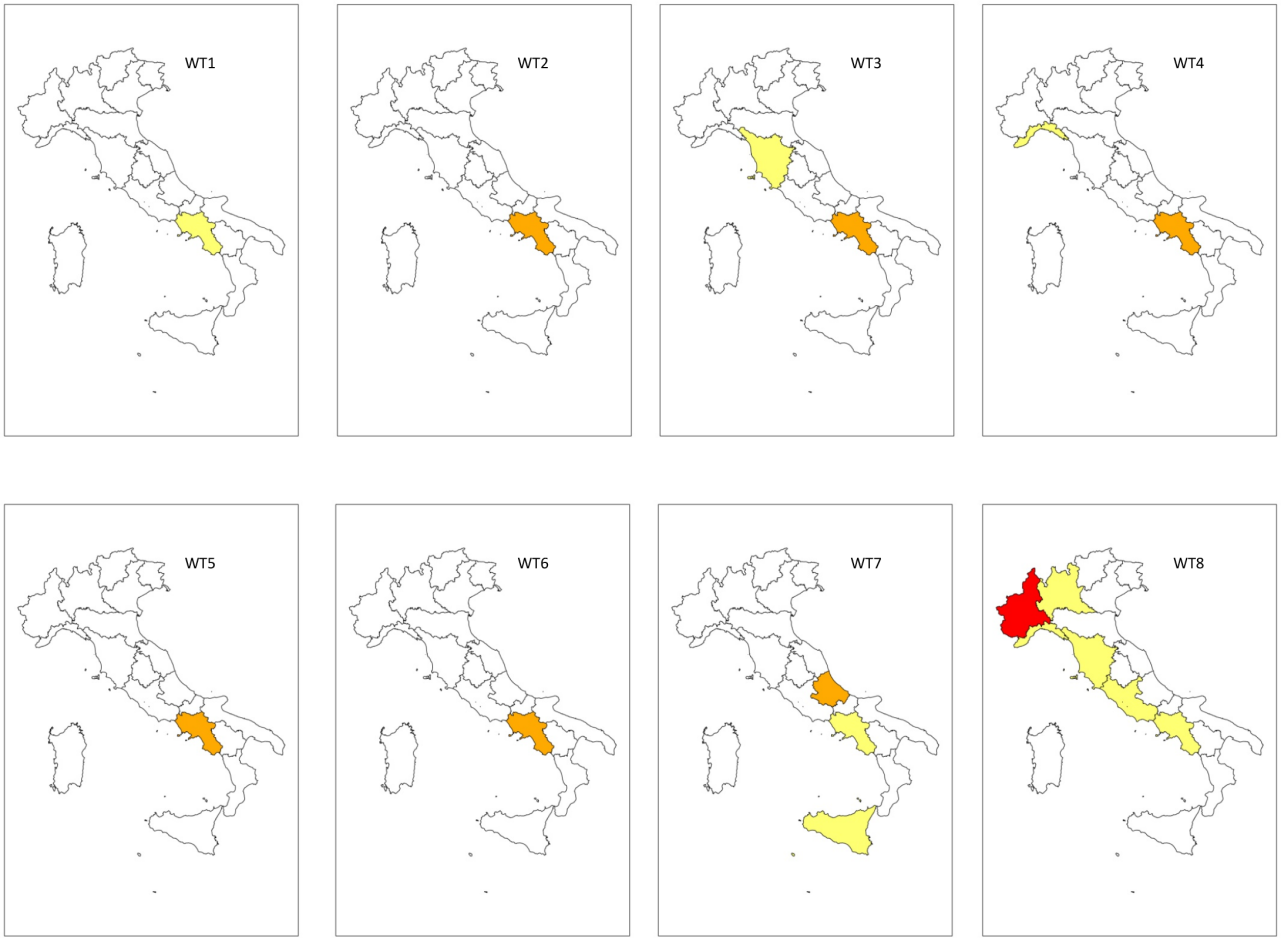
Mapping of the spring WT-related Floods and LAndslides Risk Index (WT-FLARI). WT (Weather Type). WT-FLARI levels: red = Very High (WT-FLARI > 99^th^ perc.); orange = High (95^th^ perc. > WT-FLARI > 99^th^ perc.); yellow = Moderate (95^th^ perc. > WT-FLARI > 90^th^ perc.); white = Low (WT-FLARI < 90^th^ perc.)

In spring (Fig 6), the most dangerous risk level only affected one northwestern region (Lombardy) when WT8 occurred. Conversely, the “High” risk level only occurred in southern regions, especially Campania when WT2, WT3, WT4, WT5, WT6 occurred, while for Molise was only associated with WT7. The “Moderate” risk for northern regions was only associated with WT8 (Liguria and Piedmont) and WT4 (Liguria), in central regions with WT8 (Tuscany and Lazio) and WT3 (Tuscany), and in southern regions with WT1 and WT8 (Campania) and WT7 (Campania and Sicily).

In summer (not shown), the risk was strongly downsized and localized because the perturbed Atlantic flow generally affected northern and central Europe and only occasionally Italy, above all, the northwestern regions. In particular, the “very high” risk level was only observed in Piedmont and associated with WT8. No “High” risk was observed, while the “Moderate” only involved one central region (Tuscany) when WT1 occurred.

**Fig 7:**
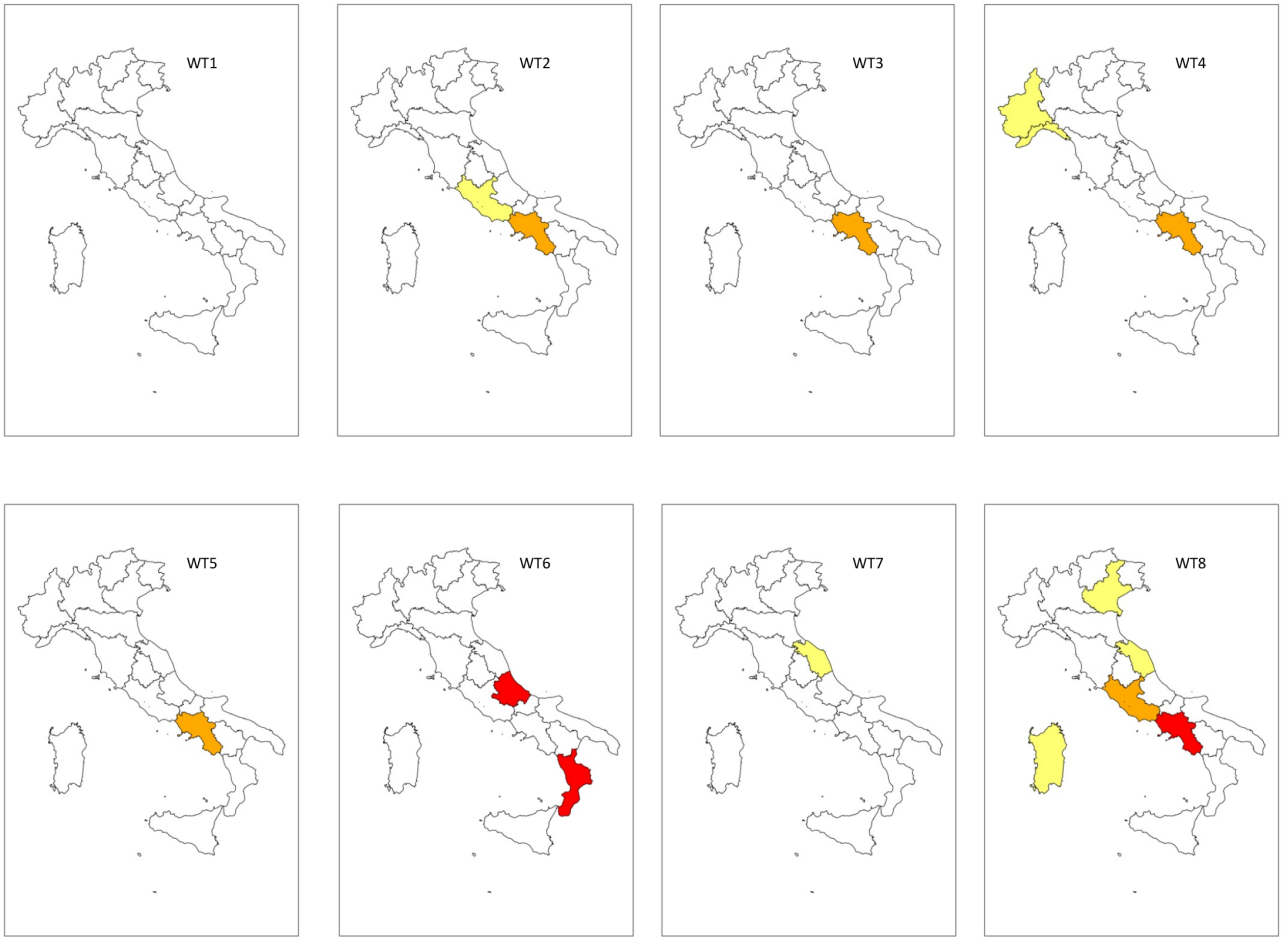
Mapping of the winter WT-related Floods and LAndslides Risk Index (WT-FLARI). WT (Weather Type). WT-FLARI levels: red = Very High (WT-FLARI > 99^th^ perc.); orange = High (95^th^ perc. > WT-FLARI > 99^th^ perc.); yellow = Moderate (95^th^ perc. > WT-FLARI > 90^th^ perc.); white = Low (WT-FLARI < 90^th^ perc.)

In winter (Fig 7), the most dangerous risk level was recorded in central and southern Italian regions when WT6 and WT8 occurred. In particular, the “Very High” risk level was associated with WT6 in Abruzzo and Calabria and with WT8 in Campania. The “High” risk was observed in one central region (Lazio) with WT8 and in one southern region (Campania) with WT2, WT3, WT4 and WT5. The “Moderate” risk was observed in northern regions associated with WT4 (Liguria and Lombardy) and WT8 (Veneto); in central regions with WT2 (Lazio), WT7 and WT8 (Marche), and in one southern region (Sardinia) with WT8.

## Discussion

The main finding of this study is that the weather type that generally determines more perturbed flows on the Italian peninsula (WT8) is associated with the highest impact in terms of damage. The greatest effects were observed during autumn, when WT8 determined a deepening of a low depression over the Gulf of Genoa with abundant rainfall in northern Italian areas. However, other weather patterns might also have important effects with variations among seasons and regions. In particular, this study revealed that the effects of each WT on a heterogeneous country such as Italy differ greatly between the northern and southern regions. The effects can even be very different between neighboring regions.

For example, in the winter period, the meteorological configurations that determined a cold easterly or north easterly flows generated rainfall primarily in the central and southern regions due to being the sites of contrast between cold air masses from the northern latitudes and warmer Mediterranean air [52, 53, 54]. For this reason, central and southern regions in particular, experienced WT-FLARI from moderate to very high risk levels. Conversely, the northern regions, despite with lower temperatures, were not affected by heavy rainfall and hence the level of risk is generally very low.

During the warm season, Italy is mainly affected by stable configurations of Azorean or African High Pressure. Moreover, in summer, but also during the intermediate seasons, the increased latent energy generated by high levels of solar radiation is potentially capable of generating strong convective precipitation even with a slight geopotential decrease [55, 56, 57]. Consequently, apparently unexpected risk conditions may be generated. This explains the occurrence of dangerous events during weather patterns characterized by high pressure. For example, weather types 4 and 5, although characterized by Azorean or subtropical high pressure in the Mediterranean basin, determined high risk conditions in some northern Italian regions during the autumn, even though few atmospheric disturbances were present.

Nevertheless, this article highlights the fact that the contribution of the “Exposure and Vulnerability layers” is essential for risk calculation. In regions where there is a high population density, a large number of rivers and little flat territory, the basic risk is generally higher. Campania is the most striking case, and in spring and winter, WT-FLARI is high for most weather types. The highest risk observed in Campania was mainly due to the great vulnerability of the territory and the high population density.

It must also be noted that this work has some limitations. In fact, the risk index was calculated according to the weather type for the event (E) day. However, the occurrence of floods or landslides was influenced by the weather conditions occurring on the days immediately preceding the event that caused the damage. Consequently, particularly prolonged rainy periods are often an aggravating factor [58, 59, 60] because pre-existing wet ground causes an increased risk of landslides. Moreover, river floods are also caused more frequently by prolonged rainy periods [61].

Further research will also study the weather types in a time lag of a few days prior to the event. This will ensure greater accuracy in calculating the risk index that will also take the persistence of the weather type into account. With the increasing reliability of seasonal forecasts, further calibration of WT-FLARI could allow for obtaining a very useful tool for preventing, or attempting to reduce, the impact of extreme rainfall events that are becoming more and more frequent [7, 62].

The World Health Organization (WHO), World Meteorological Organization (WMO), European Commission (EC), European Environmental Agency (EEA) and other important organizations encourage the development and evaluation of more effective and efficient interventions, such as early warning systems and in general, adaptation strategies to reduce negative impacts [3, 63, 64, 65, 66, 67, 68].

## Conclusions

In recent decades, the number of natural disasters caused by weather events has increased with great economic losses and a large number of deaths. For example, between 2002 and 2014 there were 293 deaths in Italy and in 2013 alone, a total of 351 landslide and flood events were recorded [22].

This study has identified a specific risk index associated with each weather type, calibrated for each Italian region and applicable to both annual and seasonal levels. The risk index represents the seasonal and annual vulnerability of each Italian region and indicates that additional preventive actions are necessary for some regions.

The result of this work represent a good starting point towards the development of a tool to support policy-makers, local authorities and health agencies in planning actions, mainly in the medium to long term, aimed at the reduction of disasters. Disasters reduction represents an important issue of the World Meteorological Organization mission [36]. Actions to be taken on the eve on an emergency will nevertheless still predominantly managed through the use of deterministic models able to better locate the phenomena, but in the medium to long term (months), the WT approach could instead make possible a better use of seasonal forecasts, which, although in gradual improving, will unlikely be able to provide deterministic forecasts, on the contrary could provide useful information on the prevailing WT. It is still necessary to reiterate that the basis of a proper land policy remains the prevention to be made in the very long term (years) taking into account the changing climate and the need to adapt infrastructures and behaviors in order to prevent the occurrence of disasters. However, this index is in the experimental stage and it doesn’t take into account the man made environmental change (for example land use, overbuilding, river flow, etc). Further investigations are required concerning the choice of "exposure and vulnerability layers" that could impact on the results. WT-FLARI will be further tested and calibrated, specifically to consider a time lag during the days immediately preceding the catastrophic event. It would also be interesting to investigate the frequency of the events and how the weather types have changed over past decades. Changes in weather patterns will be one of the principal effects of climate change and may also give rise to a different frequency of extreme weather events.
